# Contemporary Abdominal Wall Reconstruction: Emerging Techniques and Trends

**DOI:** 10.3390/jcm13102876

**Published:** 2024-05-13

**Authors:** Kaylyn Pogson-Morowitz, Denisse Porras Fimbres, Brooke E. Barrow, Nicholas C. Oleck, Ash Patel

**Affiliations:** Division of Plastic, Maxillofacial, and Oral Surgery, Duke University Medical Center, Durham, NC 27710, USAash.patel@duke.edu (A.P.)

**Keywords:** abdominal wall reconstruction, ventral hernia repair, primary fascial closure, mesh strips, component separation, biologic mesh, permanent synthetic mesh, functional abdominal wall reconstruction

## Abstract

Abdominal wall reconstruction is a common and necessary surgery, two factors that drive innovation. This review article examines recent developments in ventral hernia repair including primary fascial closure, mesh selection between biologic, permanent synthetic, and biosynthetic meshes, component separation, and functional abdominal wall reconstruction from a plastic surgery perspective, exploring the full range of hernia repair’s own reconstructive ladder. New materials and techniques are examined to explore the ever-increasing options available to surgeons who work within the sphere of ventral hernia repair and provide updates for evolving trends in the field.

## 1. Introduction

Abdominal wall reconstruction is an umbrella term for repairing a dysfunctional abdominal wall, often due to ventral hernias. Ventral hernia repair is a common procedure, with the most recent estimates of 350,000 cases performed in the United States each year with an annual incidence increase of between 1 and 2% [1,2]. The complexity of hernias presenting for repair is also increasing and requires careful operative planning for long- term success [3]. Recurrence rates after ventral hernia repair indicate that success can be challenging to achieve, with reports of recurrence rates from 15 to 40% [3]. Given the potential magnitude of some of these interventions, it is essential to consider the full breadth of options for each patient and new developments within the field.

The principal goal of reconstruction in ventral hernia repair is preventing recurrence [4,5]. The gold standard for preventing recurrence is obtaining primary fascial closure, often through close approximation reinforced with mesh [5]. Multiple methods have emerged to maximize fascial reapproximation, and mesh has proven a rich substrate for innovation. Implicated in the repair of hernias is preventing hernia complications such as bowel incarceration or strangulation, as well as optimizing the aesthetic restoration of the abdomen [6]. In addition to the surgical emergencies that hernias can precipitate, they have been shown to significantly impact patients’ quality of life and body image [6]. Interestingly, data demonstrates that patients undergoing abdominal wall reconstruction report significant increases in quality of life after repair regardless of the number of prior repairs [7]. 

Due to the complexity of many ventral hernias, a multidisciplinary team that includes plastic surgery is required to provide the most effective care [8]. The involvement of plastic surgery helps optimize tension-free closures which decreases the likelihood of recurrence [4]. In the instances in which primary closure cannot be achieved even with mesh, plastic surgery can offer component separation or free or pedicled flaps to the appropriate patients [8]. Hernias can present as challenging defects that lack an overall consensus on the best approach, and benefit from an individualized approach which plastic surgery can provide with its varied toolbox [8,9]. Multiple examples exist in the literature describing collaboration between general surgery and plastic surgery for the operative management of hernias [4,7]. Additional examples of collaboration exist between transplant surgery and plastic surgery for hernias that arise as complications after solid organ transplants [9]. The multifactorial considerations required in abdominal wall reconstruction lend themselves to a similarly diverse team to manage them.

## 2. What’s New in Primary Fascial Closure?

Primary fascial closure involves reapproximating the midline fascial edges with no remaining fascial defect. Monofilament sutures remain the conventional method for obtaining primary fascial closure. While the design of sutures and needles allows for the atraumatic passage of the material, they have a sharp leading edge and small-diameter design that concentrates forces on the tissue, which may cause damage over time [10]. The placement of mesh in a variety of planes can be used as an adjunct to primary fascial closure to provide additional reinforcement, particularly in high-tension closures; however, mesh placement requires increased tissue dissection, time for inset, and cost. Despite the benefit of prophylactic planar mesh reinforcement after primary fascial closure [11], the development of incisional and recurrent hernias remains high. A new technique using “mesh sutures” or “mesh strips” has become an increasingly popular method for achieving primary fascial closure, with unique biomechanical advantages over traditional sutures. 

The biomechanics of primary fascial closure, specifically the ultimate tensile strength (UTS) and the suture tissue interface (STI), play a crucial role in surgical outcomes [10]. The UTS is related to the properties of the suture material itself and represents the maximum stress the suture (or the repair) can withstand prior to failure. The STI is the point at which the suture meets the tissue. With standard sutures, force is especially concentrated at the STI, which can lead to tissue necrosis, suture pull-through, and ultimately surgical failure [12]. Acute failure results from suture failure or tearing through intact tissue, which can be catastrophic and may lead to dehiscence or evisceration [13]. Chronic failure manifests as incisional hernia formation, often from suture remodeling and slow migration (e.g., cheese-wiring) through the fascial edges [14]. 

The use of mesh sutures (or mesh strips) in primary fascial closure, hernia repair, and more complex abdominal wall reconstruction is a relatively recent innovation. The potential benefits of mesh sutures are numerous [10,15,16]. First, the enhanced surface area allows for improved force distribution along the STI. Additionally, this increased surface area can allow for improved tissue incorporation. Though mesh sutures have a larger amount of foreign material than a traditional suture, this may magnify the foreign body response and lead to increased scar development around the STI, resulting in stronger early wound healing. Compared to primary fascial closure with planar mesh, mesh sutures have significantly less foreign material overall and contact with the bowel, which may make this method of closure more resistant to bacterial contamination. Finally, mesh sutures require significantly less tissue dissection than is required for planar mesh placement. 

The meshed suture was first reported in the literature in 2015 by Dumanian et al. [10]. This experimental study described a novel polypropylene mesh suture with nearly identical tensile characteristics to the 0-polyprolene suture. In-vitro, the mesh suture required double the force to pull through ballistics gel. Additionally, the authors found an increased strength of early wound healing in in-vivo porcine models demonstrated by increased ultimate tensile strength, work to failure, and elasticity eight days after repair. The authors suggest filament deformation prevents the unraveling of knots and allows for more direct contact with the tissue to allow for tissue incorporation, which was confirmed via histology at eight and 90 days. A similar 2015 experimental study by Souza et al. using polypropylene mesh sutures reported better resistance to pull-through than conventional sutures in in-vivo rat models and excellent fibrocollagenous ingrowth on histologic analysis after eight weeks [17]. 

More recently, mesh suture techniques have been utilized clinically, most often for midline incisional closure and hernia repair. A 2016 study by Lanier et al. evaluated the use of mesh strips in the setting of contaminated midline closures [15]. Macroporous polypropylene mesh was cut into 2 cm strips and then used as interrupted sutures placed 1 cm apart. In their evaluation of 107 patients, 76 of which had a preoperative hernia, mesh-sutured repair yielded a recurrent hernia rate of 3.7% (n = 4). A 2018 study by Dumanian et al. investigating mesh-sutured repair of contaminated incisional hernias found comparable outcomes to established treatments [16]. In their cohort of 48 patients with hernias at least 5 cm wide, the average hernia size was 10.5 cm and 69% of patients required an anterior component separation to achieve primary fascial closure. After roughly 12 months of follow-up, 6% (n = 3) of midline hernias recurred. The authors conclude that mesh-sutured repair has a 30-day complication profile that is not worse than established treatments for complex hernia repair and suggest this technique may be appropriate for obtaining closure in more “hostile” (i.e., contaminated) fields.

Other applications of mesh-sutured repairs have been reported. In the context of open primary umbilical hernia repair, a 2023 study by Moradian et al. demonstrated favorable outcomes [18]. Their analysis of 33 patients with small (<3 cm) umbilical hernias repaired with 2 cm polypropylene mesh strips resulted in one recurrence (3%) after three years. They also collected patient-reported outcomes and found that 90% of patients were pain-free and unable to feel the knot post-operatively. A 2020 study by Kearny et al. utilized polypropylene mesh strips in the setting of traumatic iliac crest hernias. Their technique involved the repair of the hernia using 2 cm mesh strips that were passed through the fascial edge and anchored via holes drilled through the iliac crest. The authors reported no hernia recurrence at a 24-month follow-up, though only four patients were included in the case series [19]. 

In conclusion, mesh sutures or mesh strips offer an encouraging solution to overcome the limitations of conventional primary fascial closure with sutures. Though current studies are limited in size, early results are promising. Interestingly, existing studies have restricted their investigation to the use of permanent polypropylene mesh strips. Prospective studies assessing larger cohorts and longer-term outcomes, as well as exploring the use of biosynthetic mesh strips, represent exciting avenues for future research. With ongoing research and exploration of mesh sutures, the future holds exciting possibilities for improving surgical outcomes and patient experiences in abdominal wall reconstruction.

## 3. What’s New in Mesh Selection?

Mesh is used for over half of all abdominal wall reconstructions in the United States each year, making mesh selection a common deliberation [19]. The permanent synthetic mesh was initially favored for low rates of recurrence, thus reliably achieving the primary goal of abdominal wall reconstruction. However, data has increasingly emerged scrutinizing its use in the setting of long-term complications, including infection, seroma, hematoma, abscess, pain, and erosion-related complications such as bowel obstruction and enterocutaneous fistula [20]. Biologic mesh is the customary solution for contaminated fields due to its ability to revascularize and integrate with the patient’s tissue, though its ability to prevent long-term recurrence remains in doubt [21]. 

### 3.1. When to Use Biologic Mesh?

Though biologic mesh has a reputation for being expensive, it is widely recommended in guidelines for repairs in infected fields [21,22]. It does not require explant should it become exposed or the surgical site contaminated, due to its ability to support revascularization and incorporate into the soft tissue [21,22]. Most biologic meshes used are acellular dermal matrices, often of either bovine or porcine origin [21]. However, the matter of biologic versus synthetic mesh has not been settled as studies are still being published discussing the choice of mesh type, and the recurrence rate is often cited against biologic mesh [23]. In one study investigating the long-term use of biologic mesh in initial contaminated abdominal wall reconstruction, biologic mesh was demonstrated to be superior to synthetic mesh in terms of hernia recurrence [23]. However, the authors do acknowledge bias regarding mesh selection in contaminated versus noncontaminated cases [23]. Mesh infection can occur in up to 10% of ventral hernia repairs, necessitating excision of the mesh [1]. In those situations, surgeons must choose between a multi-staged repair which addresses the infected mesh in the first stage and reconstruction in the second stage, versus a single-staged approach. A recent article argues for an immediate, multi-staged approach using biologic mesh a few days after explantation, which allows operative culture antibiotic susceptibilities to result but does not allow patients to be lost to follow-up [1]. It is estimated that 21% of patients undergo the second stage of a multi-staged approach, which could be improved by performing the second stage during the same hospitalization [2]. Additionally, in comparison to a single-staged approach, any further necessary debridement could be conducted during subsequent procedures [1]. The authors do note, however, that success has been achieved in other studies by using biologic mesh in a single-staged approach [1]. They also mention that some studies found no difference in complications between using biologic and synthetic mesh single-staged approaches in contaminated fields, but that not all the patients included presented with infected mesh [1]. A recent prospective multi-center single-arm trial examined the rate of surgical site occurrence with the use of biosynthetic mesh in potentially contaminated fields in order to investigate if it would lead to early hernia recurrences in the setting of resorption [20]. They found a 26% rate within 90 days in comparison to the 6 to 55% rates reported in permanent mesh with widely variable follow-up times and study designs [20]. Overall, a variety of different studies with diverse interests have demonstrated the efficacy of biologic mesh in contaminated fields. 

A question of the mesh plane has also been linked to mesh composition. Biologic mesh in an onlay position has previously been recommended against in favor of sublay or underlay mesh due to concerns for recurrence. However, recent data has emerged exhibiting that biologic mesh as an onlay can be effective when combined with progressive tension sutures [24]. The surgical technique involved a bilateral anterior component separation and medial advancement of the rectus fascia to primarily close the defect [23]. Biologic mesh is subsequently secured in an onlay fashion using progressive tension sutures, which allows for a less invasive procedure than sublay or underlay [23]. The mean follow-up was 3.1 years and recurrence was similar to the standard of care sublay and underlay with shorter follow-up periods [23]. The authors offer a variety of explanations for the benefit imparted by the progressive tension sutures. First, the highest tension is on the most lateral suture where the tissue is most healthy, and second, the quilting both reduced dead space for potential seroma formation and assisted in the incorporation of the mesh [23]. 

Biologic mesh is additionally recommended for immunosuppressed patients such as those that make up the organ transplant or cancer patient populations [9,21]. Data has supported biologic mesh for these patients due to the lower rate of complications in comparison to synthetic mesh as biologic mesh integrates, decreasing the likelihood of infection [9]. The authors of the transplant study note that despite the higher rate of recurrence associated with mesh, their rates of complications including infection and recurrence are acceptably low when utilizing biologic mesh with component separation [9]. They demonstrate that in patients with a history of multiple, often large abdominal surgeries, and immunosuppression, their approach is effective in a mean follow-up of three years [9]. In the oncologic patient population, an advantage of biologic mesh is that exposure can often be managed with local wound care, which prevents any delay of adjuvant therapy that may otherwise have been necessary had the mesh required explantation [3]. 

### 3.2. When to Use Permanent Synthetic Mesh?

Despite the promising outcomes generated by biologic mesh, a few recent studies have challenged the assertion that permanent synthetic mesh is inappropriate for contaminated fields. In a multicenter randomized control trial, the authors compared synthetic mesh and biologic mesh in the retromusclar plane with a two-year follow-up and found that the permanent synthetic mesh significantly lowered the risk of hernia recurrence compared to the biologic mesh at 20.5% and 5.6%, respectively [25]. The two groups were equivocal in terms of complications [25], though it is possible the follow-up period was not long enough to capture the feared complications of permanent synesthetic mesh. The authors additionally emphasize the cost difference between the two types of mesh, with biologic mesh costing approximately 200 times that of permanent synthetic mesh [26]. 

Another study used a prospective clinical trial to examine the outcomes of onlay plane-permanent synthetic mesh in a clean field versus the same mesh in a known contaminated setting [26]. In the known contaminated setting, the infected mesh was removed and the new mesh was placed in a single-staged repair [26]. The authors demonstrated that the rates of surgical site occurrences were not significant between the groups and reported only one hernia recurrence in the infected mesh group after a three-year follow-up [26]. They argue that as theirs and other data support the use of permanent synthetic mesh in contaminated fields due to similar complication rates compared to clean cases, it is the correct choice given the accompanying lower rates of recurrence and lower cost [26]. 

Overall, a recent meta-analysis reported that absorbable mesh was significantly associated with recurrence in comparison to permanent synthetic mesh, as well as increased rates of surgical site infection and planned reoperation [27]. Regarding surgical site infection, the findings did not reach significance though incidence was higher in the absorbable group [27]. Though these data challenge the paradigm of avoiding permanent synthetic mesh, the authors do concede the comparison of very heterogenous studies and inclusion of both biologic and biosynthetic mesh in the “absorbable” category [27].

In the setting of recent studies increasingly supporting the use of permanent synthetic mesh, surgeons are innovating new methods of incorporating the material into reconstruction while minimizing infection risk. A publication describes using a “no-touch technique” to prevent complications and infections in the placement of permanent synthetic mesh in ventral hernia repairs [28]. The majority of these procedures were performed in the sublay plane in clean fields and the authors report lower rates of surgical site occurrence than what can be found in the literature, and no recurrence after one year [28]. Though they do not directly reference the comparative studies, their data does support the use of the “no-touch” technique similar to those often utilized in breast implant placement. Though longer-term data is not available, the principle of approaching mesh in the same way as other permanent implants is warranted. A related study by the same group, which also used the “no-touch” technique investigated outcomes of using permanent self-adhering mesh, found a shorter surgery and length of stay with no differences in surgical-site occurrences or hernia recurrence [29]. 

A third novel technique involved suturing together a permanent synthetic mesh and an acellular dermal matrix (ADM) and placing them such that the ADM was on the deeper side regardless of plane [30]. The authors reported a 7.7% recurrence rate at three years, a 15.4% surgical site occurrence rate within 60 days, and a total of two patients requiring explant [30]. The authors suggest that the physical merging of permanent synthetic mesh and ADM in this fashion merges the durability of permanent synthetic mesh with the infection resistance of ADM [30]. They additionally posit that the deeper layer protects the viscera from inflammation [30]. Another publication describes a two-stage procedure involving the creation of bilateral prelaminated tensor fascia lata flaps reinforced with permanent synthetic mesh during the first stage, and eight weeks later the flaps elevated, tunneled, and inset mesh-up during the second stage [31]. This procedure was performed on three patients who had previously failed reconstructive attempts and purposefully did not include the repair of their rectus fascia [31]. The follow-up for these patients varied between two and ten years but no recurrence or infection occurred over that time; complications included only seroma and wound dehiscence treated successfully with negative pressure wound therapy [31]. The authors attribute their success to vascularized tissue combined with the permanent synthetic mesh, as prior studies have indicated that tensor fascia latae alone are insufficient for lasting repair [31]. A final new technique for consideration is using a fixation-free permanent synthetic mesh placement in the retromuscular plane for defects 15 cm or less in width [32]. In comparison to patients who underwent repair with mechanically fixed permanent synthetic mesh, patients who received fixation-free repair had no significant difference in 30-day recurrence [32]. However, they did have significantly lower pain and abdominal wall function scores, as well as length of stay [32]. Together, these five studies demonstrate the creative approaches surgeons are taking to optimize the use of permanent synthetic mesh in hernia repair. 

### 3.3. When to Use Biosynthetic Mesh?

Biosynthetic mesh is a novel attempt to strike the balance between biologic and permanent synthetic meshes. A common composition is poly-4-hydroxybutyrate, a monofilament polymer synthesized from a monomer derived from *E. coli*, which is fully resorbed by the body in 12 to 18 months [32,33]. It has been linked to lower rates of recurrence and cost than biologic mesh while retaining the advantages derived from the absorptive nature of biologic mesh [33]. 

A recent prospective multi-center single-arm trial examined the rate of surgical site occurrence with the use of biosynthetic mesh in potentially contaminated fields in the sublay plane to investigate if it would lead to early hernia recurrences in the setting of resorption [20]. The authors demonstrated a 26% surgical site occurrence rate within 90 days in comparison to the 6 to 55% rates reported in other studies using permanent mesh with widely variable follow-up times and study designs [20]. In comparison, the surgical site occurrence rates for biologic mesh found in the literature range from 50 to 63% in similarly contaminated fields [20]. A systematic review and meta-analysis further pursued the outcomes of biosynthetic mesh in contaminated and clean fields [33]. Over six studies and 472 patients, the authors found that the rate of surgical site occurrence in contaminated cases similar to the aforementioned study was 35%, and in clean cases it was 14% [33]. Of interest, they noted a recurrence rate of 4% in contaminated cases using biosynthetic mesh in comparison to the 20.5% in biologic mesh and 4% in permanent synthetic mesh reported in the literature [33]. In clean cases, the authors discovered an 8% recurrence rate with biosynthetic mesh and in comparison reported 18% with biologic mesh and 6% with permanent synthetic mesh [33]. These data indicate that biosynthetic mesh may be more effective than biologic mesh at reducing recurrence and surgical site infection with a lower cost. 

Relatedly, biologic mesh is widely used if the intraperitoneal plane is the placement of preference given the proximity of the bowel. A recent study compared the outcomes of biologic mesh in the intraperitoneal plane to resorbable synthetic mesh in the onlay plane and found both rates of recurrence and complications to be lower in the latter group over a mean follow-up of two years [34]. Though the authors touted the longevity of resorbable synthetic mesh as the advantage over biologic mesh, the results of the study are challenging to attribute to the composition of the mesh rather than the plane of placement combined with a mesh that integrates into the patient. 

Materials and techniques for biologic, synthetic, and biosynthetic mesh continue to evolve, adding to the current armamentarium of choices and complexity of decision-making for each patient. A summary of the types of mesh discussed above, with some additional examples, can be found in Table 1. A summary of the pros and cons discussed above for each type of mesh can be found in Table 2.

## 4. What’s New in Component Separation?

Open component separation in abdominal wall reconstruction allows for autologous tissue repair with reapproximation of the midline fascia [36]. Originally described in 1961 by Dr. Young and later formalized in 1990 by Ramirez and colleagues, component separation involves transposing the rectus abdominis muscle and sheath medially. Then, the external oblique aponeurosis is cut longitudinally to allow for the rectus muscle and sheath with the attached internal oblique and transversus abdominis muscles to be advanced up to 10 cm medially at the umbilical level. Open component separation not only addresses large median defects but also avoids the use of bridging mesh [37]. This technique revolutionized the field of abdominal wall reconstruction in part because of its ability to repair large midline defects without the utilization of mesh. 

There are two variations in classic component separation: anterior and posterior [38]. Anterior component separation (ACS) involves a midline scar excision followed by skin flap mobilization [39]. The external oblique aponeurosis and muscle are divided from the inguinal region to the costal margin while the lateral border of the rectus muscle is released to mobilize the internal oblique and transversus muscles from the chest wall. Attenuated tissue surrounding the hernia is excised and the midline is ultimately closed in a single layer. This technique was used to repair large and complex ventral hernias given that prior techniques had recurrence rates anywhere between 20% and 50% [40]. While early studies showed that recurrence rates were less than 10% without the use of mesh, follow-up was limited, and long-term effects were not evaluated [30,31,32,33,34,35,36,37,38,39,40,41,42]. A significant disadvantage of ACS is wound complications from extensive lateral dissection. ACS creates large bilateral skin flaps, compromising vascularity by dividing perforator vessels. Sacrificing perforating vessels predisposes the skin flaps to ischemia and infection not to mention hematoma and seroma formation in the dead space [43]. The lateral area where the external oblique is divided is also susceptible to new hernia occurrences. Additionally, ACS is ill-equipped to address subxiphoid and suprapubic defects, and leaves limited reliable space for prosthetic reinforcement [44]. With or without mesh, the rates of complications have been estimated to be greater than 50% [45,46,47], or even up to 100% [48]. Modifications such as the preservation of perforating branches to the abdominal wall [49] or reinforcing the repair with an anterior sheath flap [50] have been shown to result in favorable outcomes. Dumanian et al., for example, developed a modified version whereby a limited lateral dissection was performed from the defect edge to expose the linea semilunaris and access the external oblique [51,52]. Simultaneously, the periumbilical perforators are preserved by dissecting in the subcutaneous plane superior and inferior to the umbilicus [51,52]. This results in a significant decrease in wound complications compared with the classic approach. Since then, new techniques have been popularized, notably posterior component separation (PCS).

In 1965, Dr. Jean Rives and Rene Stoppa crafted an approach to inguinal hernia repair and lateral ventral incisional hernias based on a retro-muscular myofascial release and sublay polyester mesh placement in the retro-rectus space [53,54]. While its use was limited to small-to-moderate hernias only, it set the stage for the technique now known as PCS. Originally described by Novitksy et al. [55], PCS involves a midline laparotomy with lysis of adhesions, followed by incision of the posterior rectus sheath and anterior dissection of the rectus muscle. The posterior rectus sheath is then incised, dividing the posterior aponeurotic sheath from the internal oblique, thereby allowing access to the plane between the internal oblique and transversus abdominis. Dissection is continued until the posterior rectus sheath is reapproximated in the midline and sutured closed. This is then followed by mesh placement in the retromuscular space and closure of the anterior rectus sheath [56]. Notably, this technique avoids subcutaneous flap elevation and minimizes the risk for flap necrosis [56]. In a small case series, only 15% of patients developed wound complications and none experienced long-term pain or abdominal wall deformities [56]. In a retrospective comparative study, wound complications and recurrence rates were significantly greater among ACS patients (48.2 versus 25.5% and 14.3 versus 3.6%, respectively) [57]. Of note, outcomes of PCS have only been reported with the use of a reinforcing prosthetic [58].

In recent years a unique variation of PCS has been introduced, known as the transversus abdominis release (TAR). As described by Novitsky et al., TAR involves first creating a retromuscular plane toward the linea semilunaris to visualize the point in which the posterior and anterior rectus sheaths come together. This helps identify and preserve the nerves innervating the rectus muscle. The posterior rectus sheath is then incised to reveal the underlying transversus abdominis, which is then divided medially. Division begins in the upper third of the abdomen where the transversus abdominis fibers are easiest to identify and separate from the underlying fascia. This allows for exposure of the plane between the transversalis fascia and the now-divided transversus abdominis muscle. Lastly, the posterior rectus sheaths on either side are reapproximated in the midline, followed by sublay mesh placement in the retromuscular space. Lastly, the anterior rectus sheaths are reapproximated to restore the linea alba [9,59,60]. Advantages of this approach include posterior rectus fascial advancement, wide dissection laterally, neurovascular supply preservation, minimal tissue undermining, and increased space for mesh sublay placement [44,61,62,63,64]. Possible explanations for the improved outcomes observed include mesh placement in a well-vascularized space created by the myofascial advancement and wide mesh overlap between the abdominal musculature and peritoneum. Additionally, the vascularized surfaces of the exposed rectus abdominis and transversus abdominis muscles minimize the risk for infection. This approach is also uniquely situated to address recurrent hernias after ACS by restoring the rectus muscles to their native orientation and reinforcing the lateral abdominal wall with mesh [65].

This technique is supported in complex abdominal defect repairs for its favorable outcomes. In a case series, while 24% of patients developed wound complications, only 7% developed major wound infections. In addition, over two years, there were only two patients that developed a recurrence [44]. A comprehensive review found complications and recurrence rates as low as 3.4% and 1.1%, respectively [66]. In a recent systematic review by Cornette et al., recurrence rates were significantly lower for this approach compared to ACS (11.9 versus 5.5%) [67]. Blair et al. similarly found that TAR was associated with few wound infections (3.2%) and recurrence rates (3%) [68]. Similarly, another study found that from over 100 patients followed, only three patients developed a superficial skin infection while one patient had recurrence [69]. Gandhi et al. found recurrence rates as low as 2.1% with no surgical skin site complications needing to be managed operatively [70]. Interestingly, a meta-analysis did not find significant differences in hernia recurrence or wound complications between patients that underwent ACS versus PCS with TAR [61]. While Krpata found that recurrence and wound complication rates favored PCS with TAR, open anterior patients had greater rates of pre-existing wound infections and diabetes which could have influenced the results [57]. While randomized controlled trials supporting this technique are not available, findings from multiple retrospective and prospective trials, systematic reviews, and meta-analyses have demonstrated the superiority of PCS with TAR in the repair of complex abdominal wall defects [57,71,72,73].

In selecting a given approach, surgeons must consider not only the characteristics of the defect itself, but also clinical factors including age, comorbidities, and hemodynamic stability. If patients have a history of prior hernia repair via an anterior approach, or history of an open abdomen, abdominal operations, skin grafting, enterocutaneous fistulas, and prior skin-only closure, PCS would be the appropriate technique to utilize [65,74]. On the other hand, PCS may not be possible in patients with a history of abdominal wall resection or extensive scarring from prior surgical interventions. Similarly, ACS would be the more suitable approach for extensive skin defects requiring elevation of the skin to allow for primary closure. The approach taken also depends on clinical judgment and preferences which differ based on surgeon experience and training.

## 5. What’s New in Functional Abdominal Wall Reconstruction?

Large composite defects of the abdominal wall involving resection or denervation of the abdominal wall musculature can have significant functional implications. Appropriate abdominal wall mechanics are critical for the flexion of the trunk, postural stabilization, and generation of intra-abdominal pressure [75]. Functional abdominal wall reconstruction is complex and has been described using both locoregional flaps and free functional muscle transfer. Additionally, progress has been made towards the neurotization of abdominal wall vascularized composite allotransplants. Table 3 summarizes the locoregional and free flap options discussed below.

### 5.1. Locoregional Flaps

Pedicled flaps from the anterior thigh including the anterolateral thigh (ALT), tensor fascia lata (TFL), rectus femoris, and vastus lateralis flap are workhorse options for full thickness defects of the abdominal wall [76]. Several of these options have also been described for functional abdominal wall reconstruction. In a 2015 study, Vranckx et al. described the “PIVA Flap” or “pedicled innervated vastus lateralis and anterolateral thigh flap”. The authors describe the elevation of a composite ALT/vastus lateralis flap with the preservation of femoral nerve motor branches to the vastus. The arc of rotation of the flap extended to the upper abdomen, even reaching the xiphoid in several cases. Significant neuromuscular retraining is required with this technique, as patients must consciously activate their thigh musculature to contract their reconstructed abdominal wall. This innervation pattern also results in the absence of abdominal “tone” at rest, leading to a persistent abdominal bulge during periods of inactivity. Despite these challenges, the authors describe significant improvements in abdominal core strength as measured by dynamometric analysis, as well as improved quality of life [77]. 

A similar approach has been described using the gracilis muscle. An early description of this technique involved the reconstruction of low, midline, abdominal wall defects primarily for complex recurrent ventral hernia [78]. A more recent description in 2018 included further mobilization of the muscle with pedicle dissection back to the profunda origin, proximal dissection of the obturator nerve, and detachment of the muscle from the pubic symphysis. This allowed the muscle to be transposed to cover the abdominal defect and secured to the xiphoid and the pubic periosteum to reconstruct the abdominal wall [79].

### 5.2. Free Functional Muscle Transfer

Free tissue transfer has also been described for functional abdominal wall reconstruction. This involves the microsurgical transfer of a donor muscle flap with vascular anastomosis to vessels in the pelvis, chest, or abdomen and donor nerve coaptation to an intercostal nerve. Re-innervation by an intercostal nerve, previously supplying the rectus abdominis muscle, is a key benefit of free functional muscle transfer as compared to pedicled flap reconstruction. This allows for the restoration of voluntary synergistic control of the reconstructed abdominal wall, obviating the need for neuromuscular retraining [80,81].

Ninkovic et al. described this technique utilizing the latissimus dorsi muscle for free functional muscle flap reconstruction of the abdominal wall. Key components of this technique involve flap harvest in the supine position, neurolysis and identification of the motor component of the intercostal nerve with a stimulator device, and restoration of the resting tension of the latissimus muscle length at the time of inset. In this 14-patient series, the authors describe excellent functional outcomes with evidence of reinnervation of the latissimus on EMG, as well as synergistic function between the donor muscle and the residual native abdominal wall at 18 months post-operation [80]. In recent years, several additional groups have reported success utilizing the latissimus muscle for functional abdominal wall reconstruction [82,83]. 

Alternative flaps such as the composite free ALT/vastus lateralis have also been described for dynamic abdominal wall reconstruction. The authors of a 2013 case series propose that a key benefit of this technique over latissimus flap reconstruction is the inclusion of the fascia lata with the flap. This provides an additional layer of structural support and may decrease the need for mesh reinforcement [84].

### 5.3. Neurotized Vascularized Composite Allotransplantaion

The highest rung on the reconstructive ladder for abdominal wall reconstruction is abdominal wall vascularized composite allotransplantation (AW-VCA) [85]. This is performed in the context of small bowel or multivisceral transplantation due to the severe abdominal fibrosis and loss of abdominal domain often encountered in these patients [86,87]. The cases of AW-VCA described in the current literature are non-innervated [88]. This is a key critique of this technique as the graft musculature is non-functional, and will inevitably atrophy without re-innervation (Reed). However, recent cadaveric and animal studies have demonstrated the feasibility of the identification of donor thoracolumbar nerves for AW-VCA, and avoidance of graft muscle atrophy with neurotization in a rat model [89,90,91]. While significant clinical challenges remain, neurotization of an AW-VCA would provide the potential for a fully functional, sensate, reconstructed abdominal wall.

## 6. Conclusions

Operative choices for abdominal wall reconstruction continue to increase and evolve as the need for this common procedure drives innovation. Ranging from primary fascial repair to proposed neurotized vascularized composite allotransplantation, hernia repair has developed its own reconstructive ladder. In addition, biomaterials and operative techniques add more layers of complexity and opportunity for creativity in surgical planning for these patients. As surgeons continue to optimize postoperative form and function in abdominal wall reconstruction, it is essential to remain abreast of the full armamentarium at our disposal for managing a complicated problem in patients for whom quality of life is so greatly impacted.

## Figures and Tables

**Table 1:** A summary of the types of mesh available for abdominal wall reconstruction [32,35].

Type of Mesh	Composition	Example
Biologic	Porcine	CollaMend, FortaGen, Permacol, Strattice, Surgisis, XenMatriX
	Bovine	SurgiMend
	Human	AlloDerm, AlloMax, Flex HD
Permanent synthetic	Polypropylene	3D Max, Atrium, Bard, Bard Soft, Marlex, Polysoft, Premilene, Prolene, Prolene Soft, Prolite, Surgipro, Trelex, Ultrapro, Ultrapro Advanced, Vitamesh, Vitamesh Blue, Vypro, Vypro II
	Polyester	Mersilene, Partietex, Versatex
	Polytetraflouroethlene	Dulex, Gore-Tex, Gore DualMesh, Gore DualMesh Plus
Biosynthetic mesh	Poly-4-hydroxybutyrate	Phasix
	Polyglactin	Vicryl
	Polyglycolic	Dexon, Gore Bio-A, Safil
	Polyethylene	Tigr Matrix

**Table 2:** A summary of the pros and cons discussed for each type of mesh available for abdominal wall reconstruction [1,9,20,21,22,23,25,26,27,33,34].

Type of Mesh	Pros	Cons
Biologic	- Does not require explant if exposed or contaminated - Incorporates into the soft tissue - Recent data with lower rates of recurrence - Recommended for immunosuppressed patients - Safe for intraperitoneal plane	- Expensive - Historically linked to higher rates of recurrence
Permanent synthetic	- Lower cost - Linked to lower rates of recurrence - Recent data supporting use in contaminated fields	- Historically not recommended for contaminated fields - Not recommended for intraperitoneal plane - Explant recommended if exposed
Biosynthetic mesh	- Incorporates into the soft tissue - Medium cost - Medium recurrence - Safe for intraperitoneal plane	

**Table 3:** Flaps described for functional abdominal wall reconstruction [76,77,78,79,80,81,82,83,84].

Flap	Blood Supply; Innervation	Advantages	Disadvantages
ALT/VL	Descending branch of LCFA; Femoral nerve branches to VL	- Minimal donor site morbidity - Arc of rotation to upper abdomen - No microvascular anastomosis required	- Neuromuscular retraining required - Lacks abdominal “tone” at rest
Gracilis	MCFA: Obturator nerve	- Straightforward flap harvest - Minimal donor site morbidity	- Limited flap size - Arc of rotation - Neuromuscular retraining required - Lacks abdominal “tone” at rest
Latissimus	Thoracodorsal artery; Thoracodorsal nerve	- Innervation from native intercostal nerves. Provides “tone” at rest. - No neuromuscular retraining - Long pedicle, reliable anatomy	- Microvascular anastomosis and nerve coaptation required - Position change may be necessary - Sacrifice of large, functional muscle

Abbreviations: ALT (anterolateral thigh), VL (vastus lateralis), LCFA (lateral circumflex femoral artery), MCFA (medial circumflex femoral artery).

## Data Availability

No new data were created or analyzed in this study. Data sharing is not applicable to this article.
